# Radiographic identification of a positive clipped axillary lymph node in a mastectomy specimen following neoadjuvant chemotherapy

**DOI:** 10.1016/j.radcr.2023.09.086

**Published:** 2023-11-14

**Authors:** Andrew Seto, Cynthia Lin, Samantha Norden, Jamie Stratton, Moira O'Riordan, Helen Pass

**Affiliations:** a Department of Surgery, Stamford Hospital, 1 Hospital Plaza, Stamford, CT 06902, USA; b Columbia University Vagelos College of Physicians and Surgeons, 630 West 168th St, New York, NY 10032, USA; c Department of Hematology and Oncology, Stamford Hospital, 1 Hospital Plaza, Stamford, CT 06902, USA; d Department of Radiology, Stamford Hospital, 1 Hospital Plaza, Stamford, CT 06902, USA

**Keywords:** Breast cancer, Clip, Mastectomy, Sentinel lymph node, Targeted axillary dissection

## Abstract

Sentinel lymph node biopsies are recommended for staging in node-positive breast cancer patients who become clinically node-negative after neoadjuvant therapy. Current guidelines support the omission of an axillary lymph node dissection if 3 negative sentinel nodes are retrieved during surgery. Consequently, the utility of routine clip placement in biopsied nodes prior to neoadjuvant chemotherapy and the necessity of targeted removal of these clipped nodes is in question. There are various methods for retrieving clipped nodes. We describe a case in which an intraoperative radiograph of a mastectomy specimen identified a clipped node that had not been localized with targeted axillary dissection in a patient with breast cancer. Pathology revealed persistent nodal positivity after neoadjuvant therapy, resulting in an escalation in care and a complete axillary dissection. We review the current literature on nodal clipping, and discuss the importance of localizing clipped nodes and the impact it can have on management.

## Introduction

The assessment of axillary lymph nodes is critical in the oncologic management of breast cancer patients. Ultrasound is frequently used to evaluate nodal involvement at the time of diagnosis. In cases of abnormal-appearing nodes on imaging, confirmatory percutaneous biopsy is performed with clip placement at the biopsy site. In breast cancer patients with biopsy-proven nodal involvement, neoadjuvant chemotherapy (NAC) is often utilized. Pathologic complete response after neoadjuvant chemotherapy ranges from 13% to 65% depending on the subtype and receptor status of breast cancer [1,2].

The National Comprehensive Cancer Network (NCCN) recommends performing a sentinel lymph node biopsy (SLNB) for staging in node-positive breast cancer patients who become clinically node-negative after neoadjuvant therapy [3]. Current guidelines support the omission of a complete axillary lymph node dissection (ALND) in patients who have at least 3 negative sentinel lymph nodes retrieved during surgery. Consequently, the utility of routine clip placement in biopsied lymph nodes prior to patients undergoing NAC and the necessity of subsequent targeted removal of these clipped nodes has been widely debated. Recent research suggests that the axillary recurrence rates are very low without nodal clipping [[4], [5], [6]] and that the pathology of clipped lymph nodes does not impact adjuvant therapy recommendations [7].

There are various techniques for retrieving clipped nodes, including radioactive seed localization, radiofrequency identification devices, localization wires, and charcoal tattooing [8,9]. Intra-operative X-ray radiographs of mastectomy specimens have yet to be described in literature when clipped lymph nodes are unable to be localized in the axilla. In this case report, we present a patient with node-positive breast cancer who underwent neoadjuvant chemotherapy that required an intra-operative radiograph of the mastectomy specimen to localize the clipped node which had not been identified with targeted axillary dissection. Furthermore, we describe how the pathology findings of the clipped node had a significant impact on our patient's management and treatment.

## Case report

**Fig. 1:**
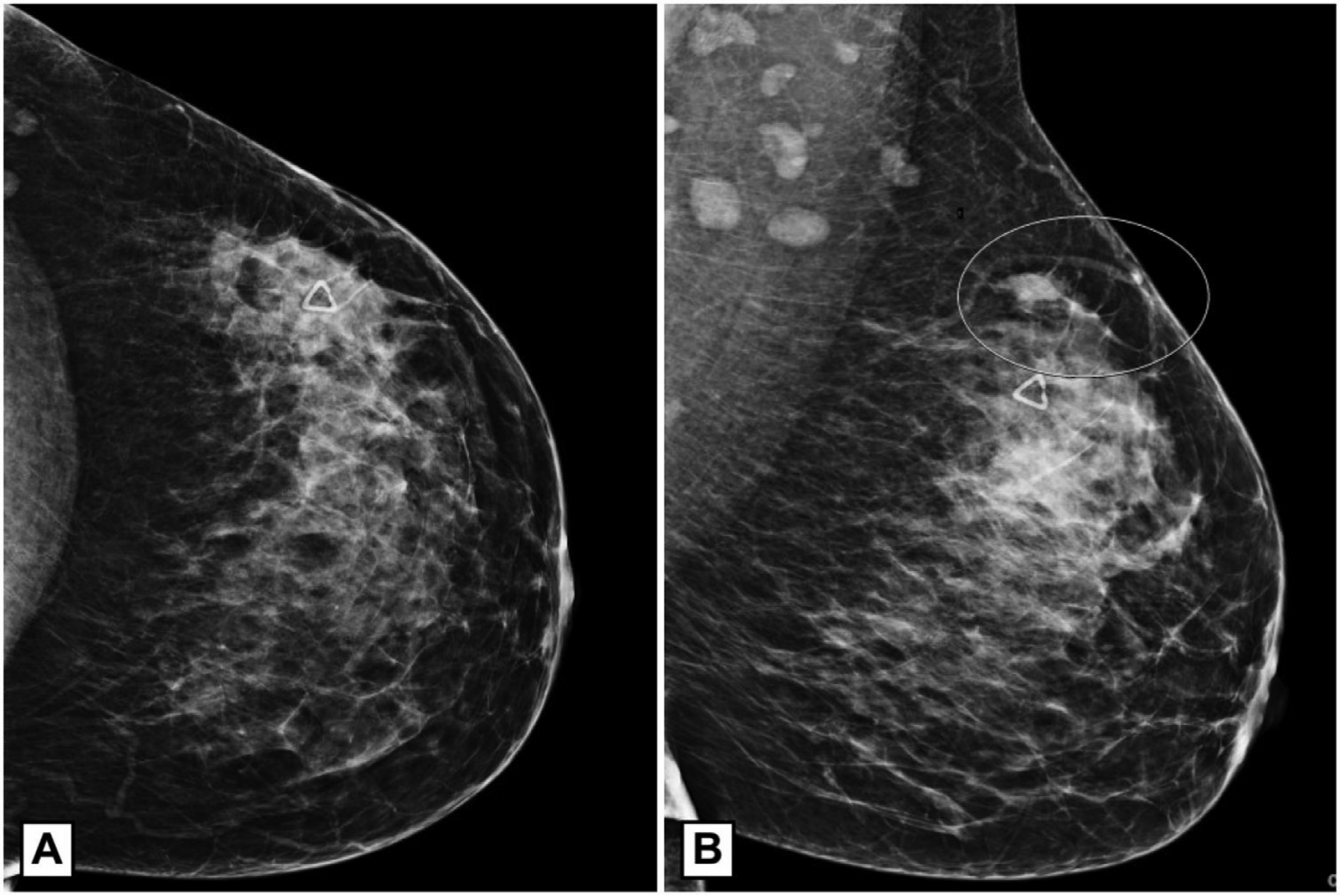
Left breast diagnostic mammogram. (A) Left breast craniocaudal view. (B) Left breast mediolateral oblique view. The breast is heterogeneously dense. A triangular marker is placed over the region of palpable concern. The oval marks an area of developing focal asymmetry in the posterior upper outer quadrant superior to the triangular marker. There are multiple prominent lymph nodes in the axilla. BI-RADS 5.

**Fig. 2:**
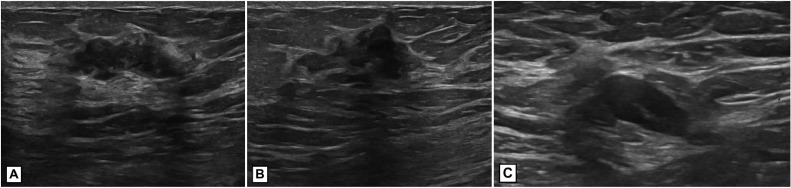
Ultrasound of left breast and axilla. Two irregular vascular masses seen on ultrasound: a 2.6 cm lesion at 1 o'clock, 12 cm from the nipple areola complex (A) and a 1.3 cm lesion at 2 o'clock, 2 cm from the nipple areola complex (B). Prominent axillary lymph node with 4 mm of cortical thickening (C).

Our patient is a 43 year-old BRCA1 gene-positive female who presented with a biopsy-proven ER+/PR–/HER2– stage IIB (T2N1M0) multicentric, left breast invasive ductal carcinoma. She initially presented with a left breast mass and axillary lymphadenopathy. A left breast diagnostic mammogram showed heterogeneously dense tissue, prominent left axillary nodes, and an area of developing focal asymmetry in the upper outer quadrant of the left breast at the area of palpable concern that was highly suggestive of malignancy, BI-RADS 5 (Fig. 1). On ultrasound at the area of mammographic concern, there were 2, irregular, vascular masses: a 2.6 cm lesion at 1 o'clock, 12 cm from the nipple areola complex and a 1.3cm lesion at 2 o'clock, 2 cm from the nipple areola complex (Fig. 2). Left axillary ultrasound revealed 5 prominent, suspicious lymph nodes with cortical thickening (Fig. 2). She subsequently underwent an ultrasound-guided core biopsy and clip placement of both breast lesions and an axillary lymph node. Pathology revealed invasive ductal carcinoma at each biopsy site.

**Fig. 3:**
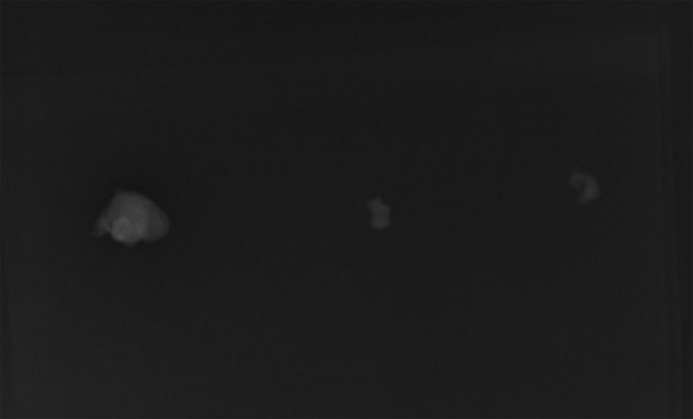
Sentinel lymph node specimens. Three axillary sentinel lymph nodes were retrieved using dual tracer mapping (Technetium-99m sulfur colloid and methylene blue dye). A clip was not identified within these nodes.

**Fig. 4:**
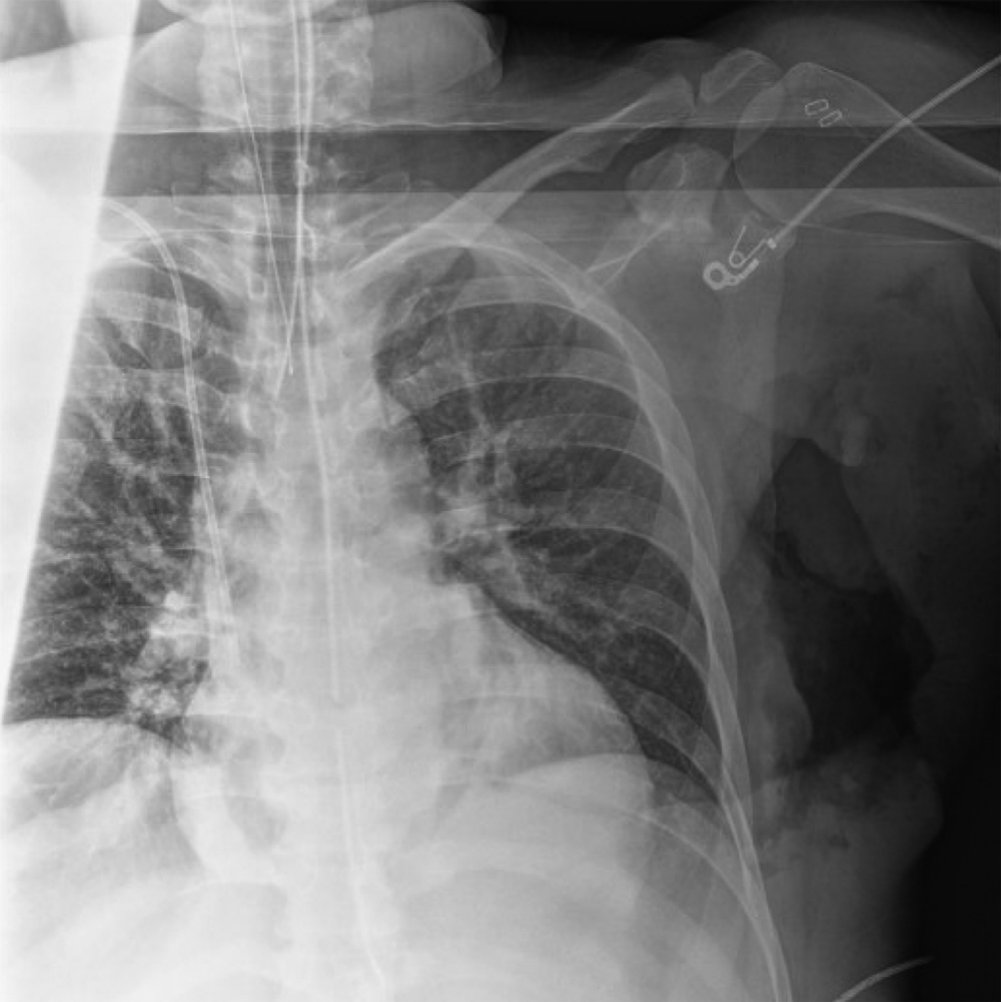
Intraoperative X-ray of chest and axilla. Intraoperative radiograph of the patient's chest and axilla did not visualize a lymph node containing a clip in-vivo.

**Fig. 5:**
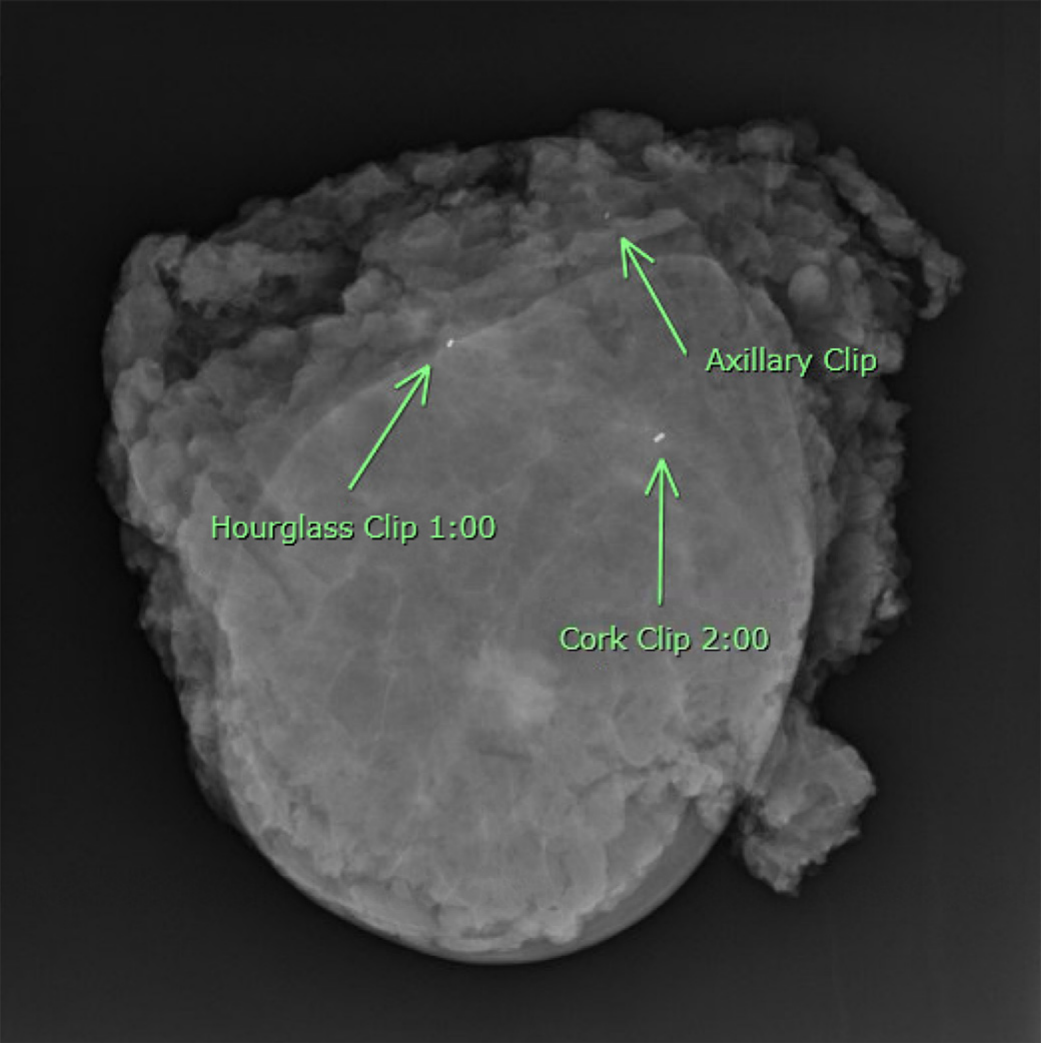
Intraoperative X-ray of mastectomy specimen. Radiograph of the mastectomy specimen showing a clip in the axillary tail as well as the 2 clips in the breast. An ultrasound-guided hourglass and cork clip was previously placed at 1:00 o'clock and 2 o'clock at the sites of the biopsy-proven invasive ductal carcinoma.

**Fig. 6:**
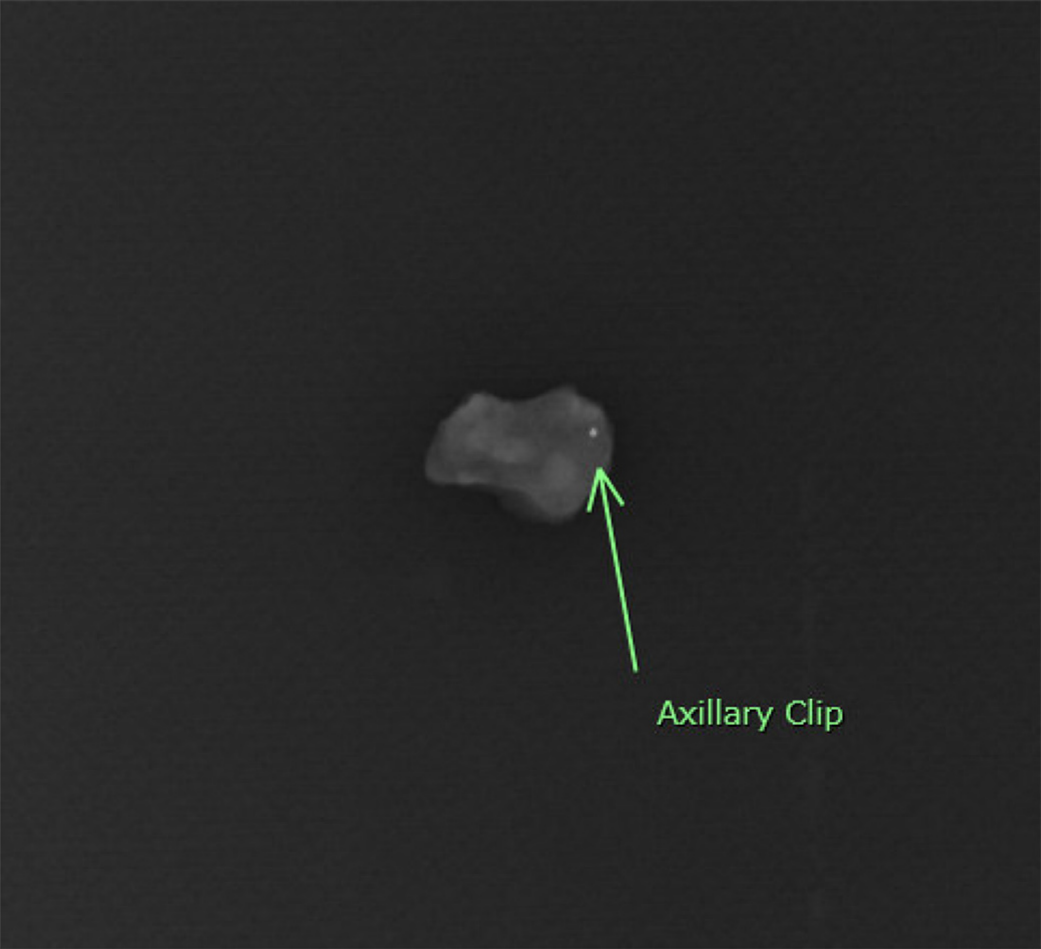
Radiograph of clipped node. The clipped node was excised from the mastectomy specimen with back table dissection and verified on radiograph.

Given the presence of nodal positivity, the patient underwent neoadjuvant chemotherapy with doxorubicin, cyclophosphamide, and paclitaxel with radiologically proven partial response. Due to her BRCA1+ status, she elected to undergo bilateral total skin-sparing mastectomies via an elliptical incision around the nipple-areola complex along with a left sentinel lymph node biopsy and targeted axillary dissection of the clipped node. The patient declined breast reconstruction. Technetium-99m sulfur colloid was injected the night before surgery and methylene blue dye was injected intraoperatively for localization of the axillary sentinel lymph nodes. The bilateral mastectomies were performed first. Interrogation of the axilla identified 3 sentinel lymph nodes: 2 blue but not radioactively hot lymph nodes, and 1 hot but not blue node. Intra-operative X-ray of the sentinel lymph nodes, however, failed to identify the localizing clip (Fig. 3). An intra-operative X-ray of the chest and axilla did not demonstrate the clip to be in-vivo either (Fig. 4). The left breast mastectomy specimen then underwent an ex-vivo specimen radiograph, which identified the clip in the axillary tail of the breast specimen (Fig. 5). The clipped node was excised with back table dissection and confirmed with a repeat radiograph (Fig. 6). The 4 lymph nodes were sent for pathological analysis. The 3 sentinel lymph nodes were negative for malignancy, but the targeted (clipped) axillary lymph node contained residual metastatic disease. In adherence to the current guidelines recommending an ALND for persistent nodal positivity after neoadjuvant chemotherapy, a complete axillary lymph node dissection was performed.

On final pathologic assessment, metastatic carcinoma with extranodal extension was identified in 5 out of the 24 total axillary lymph nodes retrieved. In the mastectomy specimen, there was a remnant focus of invasive ductal carcinoma measuring 1.5 cm, indicating partial and incomplete response to neoadjuvant chemotherapy. As a result of her significant residual burden of disease, the patient received comprehensive post-mastectomy radiation therapy to the chest wall, axilla, and supraclavicular fields. Post-operatively, the patient was also placed on adjuvant treatment with goserelin for ovarian suppression, tamoxifen, and olaparib.

## Discussion

A variety of techniques are utilized to retrieve axillary lymph nodes, and intra-operative imaging is routinely used to confirm successful removal of clipped nodes. Ultrasound-guided excision of clipped axillary nodes in node-positive breast cancer patients treated with neoadjuvant chemotherapy is safe and accurate [10]. Obtaining specimen radiographs of excised axillary nodes to confirm the presence of the localizing device is commonly employed. However, the use of an intra-operative radiograph of the mastectomy specimen to identify a clipped node when it is unable to be detected in the axilla has yet to be described in literature. To our knowledge, this is the first case report to illustrate how a radiograph of a mastectomy specimen was used to localize a clipped node that was unable to be retrieved with targeted axillary dissection in a patient with breast cancer. Furthermore, this case underscores the importance of localizing clipped nodes and the implications it can have on subsequent management.

The targeted removal of a clipped node during a sentinel lymph node biopsy has been accepted as a means to improve axillary staging. This is based on prior studies indicating that a biopsy-proven clipped node is not a sentinel lymph node in approximately 23%-35% of cases [[11], [12], [13]]. However, these studies were not optimized for a SLNB after NAC and were limited by a lack of a standardized SLNB protocol, variability in the number of sentinel nodes retrieved, and the use of single tracer lymphatic mapping. In node-positive breast cancer patients after NAC, several multi-institutional prospective studies have demonstrated that accurate nodal staging without routine clip placement is achieved with a false negative rate (FNR) <10% when sentinel lymph node biopsies are performed with dual tracer mapping (e.g., blue dye and radiolabeled colloid) and the retrieval of 3 or more sentinel nodes [[14], [15], [16]]. When a SLNB procedure is optimized in this fashion, the clipped node is a sentinel node in 88% of cases [6]. Consequently, the utility of routine clip placement and the targeted removal of clipped nodes is in question.

Recent research has supported the omission of routine nodal clipping. Bario et al. [4] found that in patients who are converted from clinically node-positive disease to node-negative after NAC undergoing SLNB with dual tracer mapping and having 3 pathologically negative sentinel nodes, the nodal recurrence rate was 0.4% at 40 months when no attempt was made to retrieve the clipped lymph node. This rate dropped to 0% with adjuvant radiation. Given the low incidence of axillary recurrence, the authors concluded that patients who have pathologic complete response on SLNB alone without retrieval of the clipped lymph node can avoid a complete ALND. Montagna et al. [6,17] demonstrated that clipped nodes are a sentinel lymph node in 88% of patients when the SLNB procedure is optimized, suggesting that failure to retrieve a clipped node should not be an indication for a complete axillary lymph node dissection. Weiss et al. [7] found that the pathology of clipped nodes did not influence post-operative adjuvant systemic therapy recommendations. Together, these findings suggest that accurate staging and treatment decisions can be accomplished with SLNB alone without the need for routine nodal clipping.

Nevertheless, current NCCN guidelines recommend that if a positive lymph node is clipped at biopsy, every effort should be made to remove the clipped node at the time of surgery [3]. Clip retrieval consistently reduces the false-negative rate of SLNB after NAC. Caudle et al. [11] demonstrated that retrieval of clipped nodes improves the pathologic evaluation of residual disease after NAC by reducing the FNR to 1.4% compared with 10.1% using SLNB alone. Dual mapper tracing did not affect the FNR. In addition, the retrieval of ≥ 3 sentinel nodes did not predict that the clipped node would be identified as a sentinel node. The only factor associated with the inability to identify the clipped node as a sentinel node was the presence of ≥4 abnormal nodes on initial ultrasound, similar to our patient who had 5 abnormal nodes seen on imaging. In the ACOSOG Z1071 trial, the FNR in patients with node-positive breast cancer who underwent both a SLNB and ALND after NAC was 12.6% [18]. Even with the addition of dual mapping agents, the FNR remained >10%. Importantly, Boughey et al. [12] showed in a subset analysis of this patient population examining the patients who had clips placed at their biopsied-proven positive nodes that clip retrieval decreased the FNR of SLNB after NAC from 14.3% to 6.8%. Other studies have produced similarly low rates with excision of clipped nodes after NAC using ultrasound-guidance and radioactive iodine seeds with a FNR of 4.1% and 7.1%, respectively [10,19].

Despite some authors recently challenging the value of nodal clipping, our case report highlights the impact it can have on management. In our patient, localizing the clipped node led to an escalation in care. The finding of residual disease in the clipped node led to the performance of a complete ALND, which revealed 5/24 nodes positive for malignancy. Without retrieval of the clipped node, a large burden of residual disease would have been left behind because the negative SLNB would have precluded an ALND. Identifying residual disease in patients after NAC is necessary for determining adjuvant therapy options [20,21]. Based on her BRCA1 germline mutation and residual disease in the breast after NAC, our patient was deemed a candidate for adjuvant olaparib, a PARP inhibitor shown to improve survival in the OlympiA trial [22]. However, her nodal positivity also expanded our treatment options by qualifying her for abemaciclib, a CDK4/6 inhibitor. In the monarchE trial, abemaciclib combined with endocrine therapy for adjuvant treatment of HR+/HER2– breast cancer patients with residual nodal disease after NAC resulted in significantly improved invasive disease-free survival and distant relapse-free survival [[23], [24], [25]]. The combination of olaparib and abemaciclib, however, has yet to be explored. Given our patient's germline mutation, high risk clinicopathologic factors, and the desire to prioritize a PARP inhibitor, olaparib was ultimately chosen. Additionally, there was no controversy in adding post-mastectomy radiation therapy with regional nodal radiation.

Overall, this case illustrates the utility of intraoperative imaging of a mastectomy specimen to localize a clipped node when it was not identified on SLNB and targeted axillary dissection. Increasingly, the finding of residual disease after NAC results in an escalation in care with demonstrated improvements in survival. Given the multitude of localization options for identifying the clipped node, the authors recommend an attempt at identification and removal. If the clipped node cannot be located and the SLNs had no residual disease, further studies evaluating the oncologic benefit of ALND is warranted.

## Patient consent

Written informed consent was obtained from the patient for publication of this case.

## References

[1] Samiei S, Simons JM, Engelen SME, Beets-Tan RGH, Classe JM, Smidt ML (2021). Axillary pathologic complete response after neoadjuvant systemic therapy by breast cancer subtype in patients with initially clinically node-positive disease: a systematic review and meta-analysis. JAMA Surg.

[2] Schmid P, Cortes J, Pusztai L, McArthur H, Kümmel S, Bergh J (2020). Pembrolizumab for early triple-negative breast cancer. N Engl J Med.

[3] National Comprehensive Cancer Network. NCCN clinical practice guidelines in oncology (NCCN Guidelines) breast cancer version 4.2023. Updated March 23, 2023. https://www.nccn.org/professionals/physician_gls/pdf/breast.pdf Accessed April 8th, 2023.

[4] Barrio AV, Montagna G, Mamtani A, Sevilimedu V, Edelweiss M, Capko D (2021). Nodal recurrence in patients with node-positive breast cancer treated with sentinel node biopsy alone after neoadjuvant chemotherapy: a rare event. JAMA Oncol.

[5] Kahler-Ribeiro-Fontana S, Pagan E, Magnoni F, Vicini E, Morigi C, Corso G (2021). Long-term standard sentinel node biopsy after neoadjuvant treatment in breast cancer: a single institution ten-year follow-up. Eur J Surg Oncol.

[6] Montagna G, Lee MK, Sevilimedu V, Barrio AV, Morrow M (2022). Is nodal clipping beneficial for node-positive breast cancer patients receiving neoadjuvant chemotherapy?. Ann Surg Oncol.

[7] Weiss A, King C, Grossmith S, Portnow L, Raza S, Nakhlis F (2022). How often does retrieval of a clipped lymph node change adjuvant therapy recommendations? A prospective, consecutive, patient cohort study. Ann Surg Oncol.

[8] Woods RW, Camp MS, Durr NJ, Harvey SC (2019). A review of options for localization of axillary lymph nodes in the treatment of invasive breast cancer. Acad Radiol.

[9] Park S, Koo JS, Kim GM, Sohn J, Kim SI, Cho YU (2018). Feasibility of charcoal tattooing of cytology-proven metastatic axillary lymph node at diagnosis and sentinel lymph node biopsy after neoadjuvant chemotherapy in breast cancer patients. Cancer Res Treat.

[10] Siso C, de Torres J, Esgueva-Colmenarejo A, Espinosa-Bravo M, Rus N, Cordoba O (2018). Intraoperative ultrasound-guided excision of axillary clip in patients with node-positive breast cancer treated with neoadjuvant therapy (ILINA Trial): a new tool to guide the excision of the clipped node after neoadjuvant treatment. Ann Surg Oncol.

[11] Caudle AS, Yang WT, Krishnamurthy S, Mittendorf EA, Black DM, Gilcrease MZ (2016). Improved axillary evaluation following neoadjuvant therapy for patients with node-positive breast cancer using selective evaluation of clipped nodes: implementation of targeted axillary dissection. J Clin Oncol.

[12] Boughey JC, Ballman KV, Le-Petross HT, McCall LM, Mittendorf EA, Ahrendt GM (2016). Identification and resection of clipped node decreases the false-negative rate of sentinel lymph node surgery in patients presenting with node-positive breast cancer (T0-T4, N1-N2) who receive neoadjuvant chemotherapy: results from ACOSOG Z1071 (Alliance). Ann Surg.

[13] Kuemmel S, Heil J, Rueland A, Seiberling C, Harrach H, Schindowski D (2022). A prospective, multicenter registry study to evaluate the clinical feasibility of targeted axillary dissection (TAD) in node-positive breast cancer patients. Ann Surg.

[14] Boileau JF, Poirier B, Basik M, Holloway CM, Gaboury L, Sideris L (2015). Sentinel node biopsy after neoadjuvant chemotherapy in biopsy-proven node-positive breast cancer: the SN FNAC study. J Clin Oncol.

[15] Kuehn T, Bauerfeind I, Fehm T, Fleige B, Hausschild M, Helms G (2013). Sentinel-lymph-node biopsy in patients with breast cancer before and after neoadjuvant chemotherapy (SENTINA): a prospective, multicentre cohort study. Lancet Oncol.

[16] Classe JM, Loaec C, Gimbergues P, Alran S, de Lara CT, Dupre PF (2019). Sentinel lymph node biopsy without axillary lymphadenectomy after neoadjuvant chemotherapy is accurate and safe for selected patients: the GANEA 2 study. Breast Cancer Res Treat.

[17] Montagna G, Morrow M (2022). ASO author reflections: do we need to clip metastatic lymph nodes at diagnosis and localize them after neoadjuvant chemotherapy?. Ann Surg Oncol.

[18] Boughey JC, Suman VJ, Mittendorf EA, Ahrendt GM, Wilke LG, Taback B (2013). Sentinel lymph node surgery after neoadjuvant chemotherapy in patients with node-positive breast cancer: the ACOSOG Z1071 (Alliance) clinical trial. JAMA.

[19] Donker M, Straver ME, Wesseling J, Loo CE, Schot M, Drukker CA (2015). Marking axillary lymph nodes with radioactive iodine seeds for axillary staging after neoadjuvant systemic treatment in breast cancer patients: the MARI procedure. Ann Surg.

[20] Masuda N, Lee SJ, Ohtani S, Im YH, Lee ES, Yokota I (2017). Adjuvant capecitabine for breast cancer after preoperative chemotherapy. N Engl J Med.

[21] von Minckwitz G, Huang CS, Mano MS, Loibl S, Mamounas EP, Untch M (2019). Trastuzumab emtansine for residual invasive HER2-positive breast cancer. N Engl J Med.

[22] Tutt ANJ, Garber JE, Kaufman B, Viale G, Fumagalli D, Rastogi P (2021). Adjuvant olaparib for patients with. N Engl J Med.

[23] Johnston SRD, Toi M, O’Shaughnessy J, Rastogi P, Campone M, Neven P (2023). Abemaciclib plus endocrine therapy for hormone receptor-positive, HER2-negative, node-positive, high-risk early breast cancer (monarchE): results from a preplanned interim analysis of a randomised, open-label, phase 3 trial. Lancet Oncol.

[24] Johnston SRD, Harbeck N, Hegg R, Toi M, Martin M, Shao ZM (2020). Abemaciclib combined with endocrine therapy for the adjuvant treatment of HR+, HER2-, node-positive, high-risk, early breast cancer (monarchE). J Clin Oncol.

[25] Martin M, Hegg R, Kim SB, Schenker M, Grecea D, Garcia-Saenz JA (2022). Treatment with adjuvant abemaciclib plus endocrine therapy in patients with high-risk early breast cancer who received neoadjuvant chemotherapy: a prespecified analysis of the monarchE randomized clinical trial. JAMA Oncol.

